# Population Structure and Spatial Distribution Pattern of *Populus euphratica* Riparian Forest Under Environmental Heterogeneity Along the Tarim River, Northwest China

**DOI:** 10.3389/fpls.2022.844819

**Published:** 2022-06-16

**Authors:** Asadilla Yusup, Ümüt Halik, Abdulla Abliz, Tayierjiang Aishan, Maierdang Keyimu, Jianxin Wei

**Affiliations:** ^1^College of Ecology and Environment, Xinjiang University, Ürümqi, China; ^2^Ministry of Education Key Laboratory of Oasis Ecology, Xinjiang University, Ürümqi, China; ^3^College of Tourism, Xinjiang University, Ürümqi, China; ^4^State Key Laboratory of Desert and Oasis Ecology, Xinjiang Institute of Ecology and Geography, Chinese Academy of Sciences, Ürümqi, China; ^5^Xinjiang Laser Radar Engineering Technology Center, Ürümqi, China

**Keywords:** LiDAR, terrestrial laser scanning (TLS), pair correlation function, kernel density, *Populus euphratica*, Tarim River

## Abstract

*Populus euphratica* Oliv. (Euphrates poplar), as the dominant tree species of desert riparian forests along the Central Asian inland rivers, plays a critical role in protecting arid land ecosystems. In recent decades, climate change and excessive water resources utilization activities have led to the environmental degradation of desert riparian forests along the Tarim River in northwest China. Understanding the forest stand structure and spatial distribution pattern provide important guidance for monitoring forest dynamics in support of sustainable management. However, few studies have examined how riparian forests stand attributes differ in response to environmental heterogeneity. In this study, terrestrial laser scanning (TLS) was applied to acquire a total of 1648 individual *P. euphratica* tree’s 3D structure attributes within 18 plots along the upper, middle, and lower reaches of the Tarim River, which included tree height (TH), diameter at breast height (DBH), crown diameter (CD), crown projection area (CPA), stand density index (SDI), age structure ratios, and spatial pattern. The results showed that the average tree segmentation and structure determination accuracies of TLS were 93.2 and 94.6%. From the upper to the lower reaches, the average TH and CD decreased by 3.8 and 0.3 m, while the DBH increased by 4.2 cm. The SDI and CPA exhibited the following order: upper reaches (454 *n* ha^–1^, 82.3%) > middle reaches (382 *n* ha^–1^, 67.3%) > lower reaches (263 *n* ha^–1^, 39.1%), the differences were significant at 0.05 level. The population age structure changed from growing population in the upper reaches to stable population in the middle and a temporarily stable population in the lower reaches. The pair correlation g(*r*) function determined random distribution pattern in the upper reaches [g(*r*) = 1.2], an aggregated pattern in the middle [g(*r*) = 3.1], and lower reaches [g(*r*) = 9.7]. The decline in groundwater depth and soil moisture increased aggregated distribution pattern (*R* = 0.67 and 0.56, *P* < 0.05) of the *P. euphratica* along the mainstream of Tarim River. The results enrich our understanding of the current development stage of *P. euphratica*, which is important for optimizing management strategies and realizing the sustainability of floodplain ecosystems.

## Introduction

Population is the key unit for the existence, adaptation, and evolution of species and is a function of individuals, communities, and ecosystems (Li and Zhang, 2015; Liu et al., 2020). Measurable variables, such as size, density, age structure, and spatial distribution patterns can provide theoretical explanations for the formation, development, and maintenance mechanism of a population, which can be employed to determine population dynamics, community succession, regeneration, mortality, and competition trends (Wiegand and Moloney, 2013; Petritan et al., 2014; Looney et al., 2018). The forest structure determines forest function, and thus maintaining the optimum state of the structural attributes is the basic premise for continuously exerting various forest functions (Pretzsch, 1997). The spatial distribution pattern is the specific arrangement of individual plants in a horizontal area, which results from population biological characteristics, intraspecific and interspecific competition, disturbances, and environmental heterogeneity (Forman and Hahn, 1980; Dessard et al., 2004; Li and Zhang, 2015; Muvengwi et al., 2018; Podlaski, 2019). This concept was specified as a quantitative indicator of generalizing complex spatial distributions of species into aggregated, random, or regular patterns (Neeff et al., 2005). In a forest stand, the detection of the distribution patterns can provide insights regarding underlying ecological processes, and such derived information is useful for scientifically guiding forest management (Pacala, 1997; Yang et al., 2019). Béland et al. (2003) analyzed the *Pinus banksiana* forest distribution pattern index and concluded that interspecific competition reduced the stand density. Kint (2005) studied the transformation of *Pinus koraiensis* into mixed forests and showed that mixed forests had a growing trend, the distribution pattern had shifted from aggregated to random distribution, and the tree size diversity had increased. Vandekerkhove et al. (2018) investigated the beech forest and found that the aggregation consequences of trees decreased with increasing tree age, and distribution gradually became more random in a less disturbed forest. Kane et al. (2015) found that water balance and topography were multiple factors that affect forest structure patterns. Courbaud et al. (2001) simulated the effects of different thinning methods on *Picea abies* forest spatial structure and concluded that selective cutting increases the tree spacing, conducive to the growth of large trees. In contrast, thinning in groups of trees improves light conditions and benefits the growth of medium trees and sapling renewal. Arruda et al. (2016) found that inundation and fire hazards can shape the structures of riparian forests. Overall, forest spatial distribution patterns can provide fundamental implications for adjusting the unreasonable forest structure to the best state and sustainably exerting ecological functions.

The *Populus euphratica* Oliv. (syn. *Populus diversifolia* Schrenk; Euphrates poplar) is a broad-leaved deciduous tree species that form the riparian forest ecosystem in inland river basins across Central Asian arid regions (Westermann et al., 2008; Thevs et al., 2012; Thomas, 2014; Lang et al., 2016) which is highly drought and salinity tolerant (Zeng et al., 2009; Cao et al., 2020; Keram et al., 2021). The Tarim River Basin, NW China, holds approximately 54% of global *P. euphratica* forests (Hai et al., 2006; Ling et al., 2015). In this region, the *P. euphratica* as a dominant tree species of the natural riparian forests, provide essential ecosystem services in arid and semi-arid regions, stabilize vulnerable ecosystem by preventing natural disasters such as sandstorms, soil erosion, and desertification (Chen et al., 2014; Betz et al., 2015; Keyimu et al., 2017b; Mamat et al., 2018, 2019; Halik et al., 2019). However, these desert poplar forests are experiencing a decline and have even disappeared due to the reduction of Tarim water resource (Zhang et al., 2019). Diversions of the Tarim River for irrigation purposes have reduced water flow and flooding frequency, contributing to forest degradation (Thevs et al., 2008; Xu et al., 2009; Deng, 2016; Chen et al., 2019). Therefore, systematic research on the population structure attribution of *P. euphratica* forests is essential in understanding the response characteristics of this species under heterogenic environments. This will consequently allow us to optimize the conservation and restoration strategies of these forests (Yu et al., 2021).

Over the past decade, numerous studies examined the effects of natural flooding disturbance on the rising groundwater level, plant diversity, survival, and growth rate of the *P. euphratica* population within different environmental conditions (Keyimu et al., 2017a; Thomas et al., 2017; Zeng et al., 2019; Thomas and Lang, 2020). The *P. euphratica* occurrence has decreased with increasing distance from river channels (Halik et al., 2006; Aishan et al., 2013; Zhang et al., 2019). The groundwater depth is a key factor affecting riparian forests growth and development (Keram et al., 2019; Gai et al., 2021; Shi et al., 2021). In the upper reaches of the Tarim River, the *P. euphratica* population age structure follows a pyramid structure with an increasing trend (Han et al., 2013, 2014, 2017), while in the middle and lower reaches, the population structure exhibits an inverted pyramid shape, with a declining trend (Xu et al., 2016; Zhou et al., 2018). Han et al. (2016) and Shi et al. (2021) revealed that the interspecific and intraspecific competitive relationship of *P. euphratica* gradually increased with the growth stage and led to reduce its population density to ensure survival. Zeng et al. (2019) studied different age class tree distribution and found the spatial patterns of young and old *P. euphratica* trees in the upper reaches of the Tarim River exhibit aggregated and random distribution. Miao et al. (2020) investigated the spatial distribution of live and dead trees of *P. euphratica* in the upper reaches and found that they showed a positive association in the juvenile plot. Most of the studies focused on investigating the spatial distribution of *P. euphratica* in the upper or middle reaches of the Tarim River *via* traditional field survey methods (Bai et al., 2013; Xu et al., 2016; Zhou et al., 2018). However, with the expansion of water demand in the upper reaches of the Tarim River, the water supply has been decreased in the middle and lower reaches (Chen et al., 2019; Zeng et al., 2019). To our knowledge, there is no investigation which comparatively analyzed the *P. euphratica* stand structural attributes, population dynamics, and distribution patterns across heterogenic environmental conditions in the upper, middle, and lower reaches of the Tarim River. One of the reasons is that the traditional field survey methods need high labor intensity and time consuming for acquiring multiple stand forest structural parameters. As an effective technique for measuring detailed tree attributes in forests, terrestrial laser scanning (TLS) is widely used in forest surveys to accurately obtain 3D structure information of forests (Su et al., 2016; Guo et al., 2018). However, TLS technology has not been applied in measuring desert riparian forests along the Tarim River.

In the present study, we have used TLS for the first time to obtain the stand structure and spatial distribution pattern of *P. euphratica* riparian forest, which improves the efficiency and accuracy of forest inventories. We determined and comparatively analyzed the population structure and spatial distribution patterns of *P. euphratica* along the upper, middle, and lower reaches of the Tarim River. The objects are to clarify the population structure, survival status, and future development trend of *P. euphratica* riparian forest. We aim to (1) determine the accuracy of TLS in detecting the tree structure of *P. euphratica*; (2) clarify the differences in tree morphological parameters of *P. euphratica* in the upper, middle, and lower reaches of the Tarim River; (3) identify the population structure and spatial distribution patterns of *P. euphratica* in different river transects; (4) explain the effect of groundwater, soil moisture, salinity, and pH values on the corresponding distribution patterns of riparian forests. Our study enriches the understanding of the development stage of *P. euphratica* forests under heterogenic environmental conditions.

## Materials and Methods

### Study Area

**FIGURE 1:**
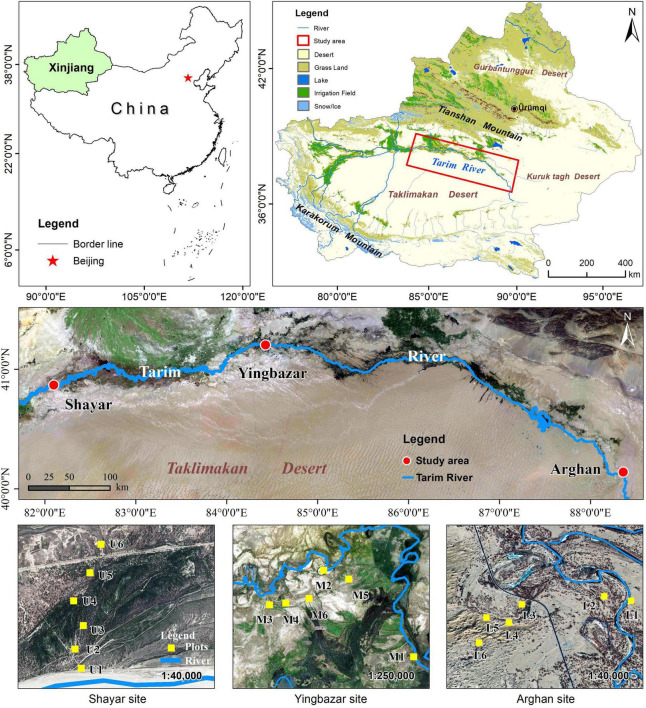
Map of the study area and location of sampling sites.

The study was conducted at the Shayar site on the upper (U), at the Yingbazar site on the middle (M), and at the Arghan site on the lower (L) reaches of the Tarim River (40.57°N–40.08°N, 82.22°E–88.21°E, ASL 1000–800 m) within the northern edges of Taklamakan Desert, northwest China (Figure 1). This area is characterized by a typical continental temperate arid desert climate (Kottek et al., 2006). The mean annual temperature, precipitation, and evaporation range from 9 to 11°C, 50 to 80 mm, and 2500 to 3000 mm, respectively (Aishan et al., 2016; Keyimu et al., 2017a). There is low precipitation, and the main water source is the Tarim River which is derived from Tianshan and Karakorum Mountain’s snow meltwater and rainfalls. According to the Food and Agriculture Organization (FAO) classification of the world’s soils, Fluvisols, Gleysols, Solonchak, and Arenosols occur as the soil types in the study area (Thevs et al., 2008). The Tarim River is fringed with riparian forests, and the growing season is from April to October. The main vegetation species are *P. euphratica*, *Tamarix* spp., *Phragmites australis*, *Alhagi sparsifolia*, *Karelinia caspia*, *Lycium ruthenicum*, *Halimodendron halodendron*, *Apocynum venetum*, *Hexinia polydichotoma* (Kurban et al., 2019), among these, the *P. euphratica* is the dominant tree species (Zeng et al., 2009; Thomas et al., 2016).

### Field-Data Collection

**FIGURE 2:**
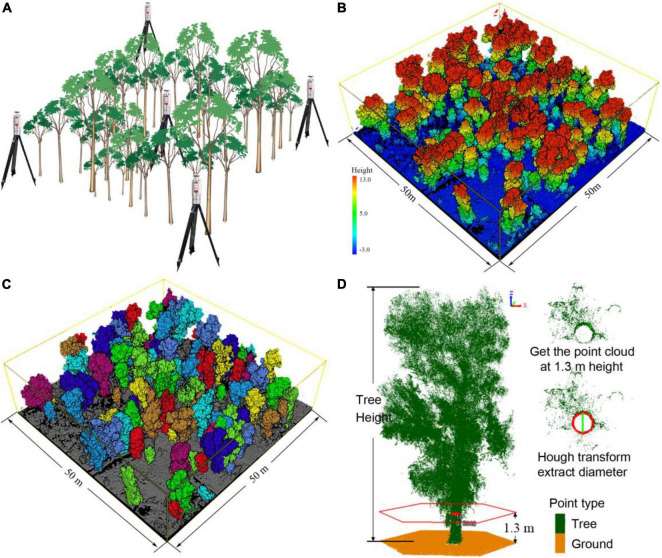
**(A)** Schematic diagram of the TLS scanning layout, located at the center point and four corners of each plot. **(B)** Original TLS multi-station combined point cloud data of plot U5. The Change in color from blue to red indicates an increase in altitude. **(C)** Individual *P. euphratica* tree identification results which represented by different colors. **(D)** Individual tree point cloud data, where green points indicate tree. The highest green point indicates the tree height (TH) value, and the red circle is the position of the cross-section used to obtain the diameter at breast height (DBH) value.

A total of 18 sample plots were established in the upper, middle, and lower transects of the Tarim River. At each transect, six *P. euphratica* plots (U1–U6, M1–M6, L1–L6) with a quadrat area of 50 m × 50 m were set up maintaining a distance of 20, 200, 400, 600, 800, and 1000 m from the river channel, respectively (Table 1). Considering the *P. euphratica* forest distribution area, and the river line changes complexity, the plots cannot be arranged following one line, particularly at the middle reaches. But we ensured that the straight distance of each plot from the river channel had an equidistantly increased pattern (Figure 1). In each plot, *P. euphratica* is the only tree species. Considering that seedlings below 1.5 m were difficult to distinguish from shrubs, affecting the segmentation of individual trees, and thus only the trees taller than 1.5 m were identified using TLS. The biometric parameters (TH, tree height; DBH, diameter at breast height; CD, crown diameter) were subsequently determined (Figure 2). TLS data was collected in May 2020 under wind speeds lower than 3 m/s (Yusup et al., 2020). The average groundwater depth was obtained from groundwater monitoring wells set up in each section by the Tarim River Basin Administration Bureau. Soil moisture, salinity, and pH values were measured in each plot at 50 cm soil depth using Hydra soil moisture sensor (Stevens, Co. Ltd., Horn, Germany).

**TABLE 1:** Basic information of the *P. euphratica* forest plots.

Location	UAV photos (50 m altitude)	Plots-ID	Distance from river channel (m)	Altitude (m)
	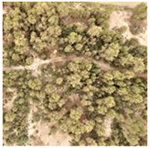	U1	20	980
Upper reaches		U2	200	977
Xayar County		U3	400	975
Ermuchang		U4	600	983
40°56′N, 82°20′E		U5	800	977
		U6	1000	969

	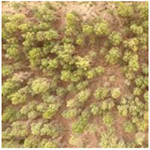	M1	20	926
Middle reaches		M2	200	908
Luntai County		M3	400	921
Yingbazar		M4	600	927
41°12′N, 84°16′E		M5	800	943

		M6	1000	909
	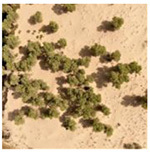	L1	20	815
Lower reaches		L2	200	807
Ruoqiang County		L3	400	822
Arghan		L4	600	813
40°8′N, 88°21′E		L5	800	818
		L6	1000	827

*U, M, and L denotes plots in the upper, middle, and lower reaches, respectively.*

#### Terrestrial Laser Scanning Data

Terrestrial laser scanning is one of the light detection and ranging (LiDAR) technologies that collect point cloud data at the speed of light and information on the flight time of a laser pulse (Guo et al., 2018; Calders et al., 2020). Unlike traditional remote sensing methods, TLS laser pulse measurements can describe the tree crown and obtain 3D structure information of forests in detail, avoiding time-consuming and labor-intensive data collection (Means et al., 2000; Su et al., 2016; White et al., 2016). We employed a Riegl VZ-1000 TLS (Riegl, Co. Ltd., Horn, Austria) for the measurements. TLS is installed on a 1.5 m high tripod when conducting data collection. The scanning range of the device was set to a vertical angle of 30°–130°, a horizontal angle of 0°–360°, and a scanning distance of 450 m; the laser pulse intensity was 300 kHz, produced a discrete point dataset containing a single laser pulse, the average point densities of 5000 points/m^2^; spending 12 min/site for each individual scanning. In order to obtain full coverage for the vegetation three-dimensional surface point cloud data, we set up five TLS scanning positions for each plot (Figure 2A). The coordinate station adjustment of the point cloud data was carried out using a Riscan Pro (V. 2.7, Riegl, Co. Ltd., Horn, Austria). Figure 2B presents the TLS pre-processing data, with a standard deviation less than 0.01 m. Structural tree parameters were acquired using LiDAR 360 (V. 4.0, Green Valley, Co. Ltd. Beijing, China) *via* a bottom-up approach to identify individual trees (Tao et al., 2015; Figure 2C). A total of 1648 individual *P. euphratica* trees were detected *via* the TLS scans in all plots (681 trees in the upper reaches; 573 trees in the middle reaches; and 394 trees in the lower reaches). The coordinates and biometric parameters (TH, DBH, and CD) of all free-standing tree stems were determined at a resolution ≤ 1 cm (Figure 2D).

#### Verification of Terrestrial Laser Scanning Data Accuracy

**FIGURE 3:**
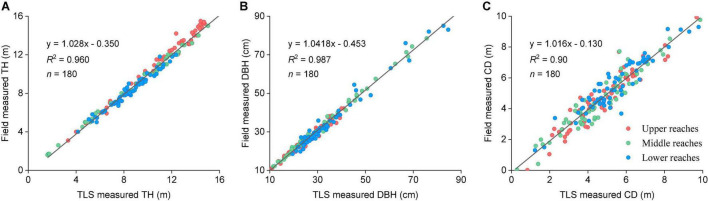
**(A)** Linear statistical regression analysis of TH results. **(B)** Linear statistical regression analysis of DBH results. **(C)** Linear statistical regression analysis of CD results between TLS and field data. Blue, red, and green points indicate the values acquired from the upper, middle, and lower reaches.

To validate the accuracy of the TLS data, we selected 10 individual trees in each plot and manually measured the tree biometric parameters of H, DBH, and CD by using the Blume-Leiss altimeter (Harbin Optical Instrument Factory Ltd, Heilongjiang, China), DBH rulers, tape measure. At the same time, we have recorded the geographical coordinates by using a Real-time kinematic (RTK) positioning system (Hi-Target Navigation Tech Co. Ltd. Guangzhou, China). Linear correlation analysis was subsequently performed between the TLS and manually measured data using Origin (V. 9.8, Origin Lab., Co. Ltd. Massachusetts, MA, United States). From the upper to lower reaches, the average individual tree segmentation accuracy of the TLS point clouds in three transects were 89.9, 94.6, and 96.2%, respectively. The average segmentation accuracy was 93.2%. The TLS-determined tree parameters were consistent with the field measurements, with coefficients (*R*^2^) of 0.96, 0.98, and 0.90, respectively (Figures 3A–C).

#### Population Division and Stand Parameters Calculation

The DBH values determined from the TLS survey were classified into 21 groups, each consecutive group separated by 4 cm, according to Keyimu et al. (2017b). Trees with DBH values greater than 80 cm were relatively less abundant and were thus grouped. We adopted the method of replacing age grade by DBH grade (Yang et al., 2019). The 21 groups were then classified in four grades according to the following: young trees (1 cm < DBH ≤ 15 cm); near-mature trees (15 cm < DBH ≤ 30 cm); mature trees (30 cm < DBH ≤ 50 cm); old trees (50 cm < DBH) (Ling et al., 2015). The total number of trees (*n*) was derived from the TLS dataset; the stand density index (SDI) was calculated by dividing the number of trees to plot area; and the total crown projections area (CPA) was determined as the percentage of the canopy cover in the whole plot.

### Identification of Spatial Distribution Pattern

#### Pair Correlation Function

In order to analyze the observed small-scale sequence of the mapped tree points, we determined the pair correlation function value g(*r*) (Wiegand and Moloney, 2013), which is related to the derivative of Ripley’s K(*r*) function (Ripley, 1976). Ripley’s K(*r*) function divides the expected number of points occurring within distance *r* of the specific point by intensity λ of the pattern:


(1) K(r)=λ-1n-1∑i=1n∑j=1nWij-1Ir(dij<s)

where, *r* is the distance scale; λ is the average point density; *n* is the number of trees in plot; *d* is the Euclidian distance between points *i* and *j*; *s* is the spatial lag; *I*_*r*_ is an indicator function which is 1 if *d_*ij*_* ≤ *r*, and 0 if *d_*ij*_* > *r*; and *W*_*ij*_ is an edge correction applied for trees close to the plot periphery edge.

The g(*r*) is a probability density function that can be interpreted as the neighborhood density. Unlike K(*r*), g(*r*) has the advantage of being non-cumulative and is also more sensitive to small-scale effects (Muvengwi et al., 2018). Thus, we employed the g(*r*) function to analyze the spatial distribution patterns of each tree (Wiegand and Moloney, 2013):


(2) $$\begin{array}{c} \text{g}\,\left(r\right)=\frac{1}{2\,\pi \,r}\,\frac{\mathrm{dK}\,\left(r\right)}{d\,\left(r\right)} \end{array}$$

Assuming that the complete spatial randomness (CSR) null model condition holds, g(*r*) = 1, indicate random distribution pattern, g(*r*) < 1 indicate regular distribution pattern, while values of g(*r*) > 1 indicate aggregated spatial distribution patterns (Carrer et al., 2018). The CSR model assumes that any point in the pattern has an equal probability of occurring at any location within the plot, with a homogeneous environment. All statistics were performed using the *Programita* (V. 2018). Here, the grid size was set as 1 m^2^ with a ring width of 1 m and a range from 0 to 25 m (Yang et al., 2019). The CSR null model was evaluated 199 times to generate a 99% confidence interval using Monte Carlo simulations. g(*r*) values above, below, and in the middle of the CSR confidence interval indicated aggregated, regular, or random distribution patterns, respectively (Svátek et al., 2018).

In order to determine the spatial pattern more accurately, we also employed the Average nearest neighbor index, Hopkins–Skellam index, David–Moore index, Morisita’s index, and Kernel intensity, respectively. The specific explanations are as follows:

#### Average Nearest Neighbor Analysis

The average nearest neighbor (ANN) ratio, which is identical to the Clark-Evans aggregation index, is quantified the attributes and correlations among points. The ANN measures the distance between each feature location and the location of its nearest neighbor, which is calculated using the formula:


(3) $$\begin{array}{c} \text{ANN}=\frac{\frac{1}{n}\,\sum_{i=1}^{n}d_{i}}{\frac{1}{2}\,\sqrt{A/n}} \end{array}$$

where, *d*_*i*_ is the distance between tree *i* and its nearest neighbor; *n* is the total number of trees; and *A* is the plot area; ANN index values less than, close to, and greater than 1 indicate aggregated, random, and regular spatial patterns, respectively (Yang et al., 2019). The ANN ratio was determined in ArcGIS (V. 10, Esri, Co. Ltd. California, CA, United States).

#### Hopkins–Skellam Index

The Hopkins–Skellam index (HSI), also called the coefficient of aggregation, the method depends on linear measurements between random points and adjacent individuals and between adjacent pairs of individuals (Hopkins, 1955), which is calculated using the formula:


(4) HIS=∑i=1NPi2∑i=1NIi2

where, *N* is the sample size; *P*_*i*_ is the distance from a point chosen at random to its nearest individual; *I*_*i*_ is the distance from an individual chosen to the nearest individual. The HIS index values are less than, close to, and greater than 1 indicating regular, random, and aggregated spatial patterns.

#### David–Moore Index

The cluster size of the David–Moore index (DMI) is employed (David and Moore, 1954), which is intensively influenced by population density:


(5) $$\begin{array}{c} \text{DMI}=\frac{\sum_{i=1}^{n}\left(x_{i}-\bar{x}\right)}{\bar{x}\,\left(n-1\right)} \end{array}$$

where, $\bar{x}$ is the mean value of population abundance; *n* is the number of quadrats; *x*_*i*_ is the number of trees. DMI index values less than, close to, and greater than 1 indicates regular, random, and aggregated patterns.

#### Morisita’s Index

The calculation of the Morisita’s index (*I*_δ_) is as follows:


(6) $$\begin{array}{c} {\text{I}}_{\delta }=\text{n}\,\frac{\sum_{i=1}^{n}x_{i}\,\left(x_{i}-1\right)}{N\,\left(N-1\right)} \end{array}$$

where *N* is the total number of trees; the index *I*_δ_ 1 values less than, close to, and greater than 1 indicates regular, random, and aggregated for a random distribution, *I*_δ_ 1 for aggregated spatial patterns, respectively (Bunyavejchewin et al., 2003). To test the significance of these distribution pattern indexes deviating from the Poisson distribution, a *t*-test is performed.

#### Kernel Intensity Mapping

Intensity maps can be created using the location data of samples *via* Kernel estimation. The contribution of each data point is smoothed out from a single point into a region of surrounding space (we used a circle with a 5 m radius in the present study). Aggregating the individually smoothed contributions produces an overall map of the data structure. *Programita* was employed to derive and display the intensity maps (Wiegand and Moloney, 2013).

### Relationship Between Distribution Patterns and Abiotic Factors

*Populus euphratica* forests in the study area are mainly distributed in the flat terrain areas in the riparian zone (Table 1) and grow under the same exposure condition. Previous research results indicated that groundwater condition (Cao et al., 2020; Thomas and Lang, 2020) and soil physicochemical properties (Han et al., 2016; Zhang et al., 2019) were the main environmental factors affecting the distribution of desert riparian forests. Therefore, we selected groundwater depth (GD), soil moisture (SM), soil conductance (SC), and pH values as abiotic factors correlated with the spatial distribution pattern index by the Pearson correlation analysis method. Stata (V13.0, Stata, Co. Ltd. Texas, TX, United States) software was adopted for calculation.

## Results

### Terrestrial Laser Scanning-Based Tree Structural Information

The numbers of total and young trees, the SDI, TH, and CPA values significantly reduce from the upper to the lower reaches of the Tarim River (*p* < 0.05). In contrast, the number of old trees and DBH and CD values peaked in the middle transect. Mature and near mature tree numbers were the highest in the lower reaches (Table 2).

**TABLE 2:** Structural parameters of *P. euphratica* in different transections of the Tarim River.

River transect	Num. of trees (*n*)	Young trees (%)	Near-mature trees (%)	Mature trees (%)	Old trees (%)	Average				
						SDI (n ha^–1^)	TH (m)	DBH (cm)	CD (m)	CPA (%)
Upper reaches	681^a^	29^a^	43^a^	23^a^	5^a^	454^a^	10.2 ± 2.4^a^	24.6 ± 0.1^a^	4.5 ± 0.8^a^	82.3^a^
Middle reaches	573^a^	18^b^	45^a^	25^a^	11^b^	382^b^	9.29 ± 1.6^a^	31.4 ± 0.1^b^	4.7 ± 0.7^a^	67.3^b^
Lower reaches	394^b^	14^c^	48^a^	29^b^	9^b^	263^c^	6.39 ± 1.2^b^	28.8 ± 0.2^c^	4.3 ± 0.8^a^	39.1^c^

*Different alphabets in the same column indicate significant differences (p < 0.05).*

### Population Structure of *Populus euphratica* in Different Transects

**FIGURE 4:**
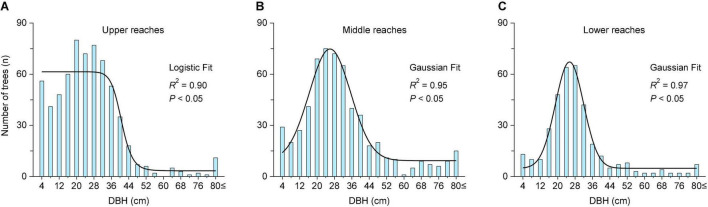
The number of *P. euphratica* trees in different DBH classes in the three transects of the Tarim River. The black line represents the curve fit of the nonlinear regression analysis. The results were significant at the 0.05 level.

The population structure of *P. euphratica* trees in the upper reaches exhibits a reversed–*J* distribution (logistic fit, *R*^2^ = 0.90) (Figure 4A). The near-mature trees occupy the highest ratio (43%), followed by young trees (29%), mature trees (23%), and then old trees (5%) (Table 2). The number of young trees (*n* = 197) is almost six times that of old trees (*n* = 34). The population structure of the middle and lower reaches follows a Gaussian fit (*R*^2^ = 0.95 and 0.97) (Figures 4B,C), where near-mature trees occupy the highest ratio (45 and 48%), followed by mature trees (25 and 29%), young trees (18 and 14%), and old trees (11 and 9%) (Table 2). Young and near mature trees occupy a higher ratio in the middle reaches compared to the lower reaches (29%, 23%).

### Spatial Distribution Pattern of *Populus euphratica* in Different Transects

**FIGURE 5:**
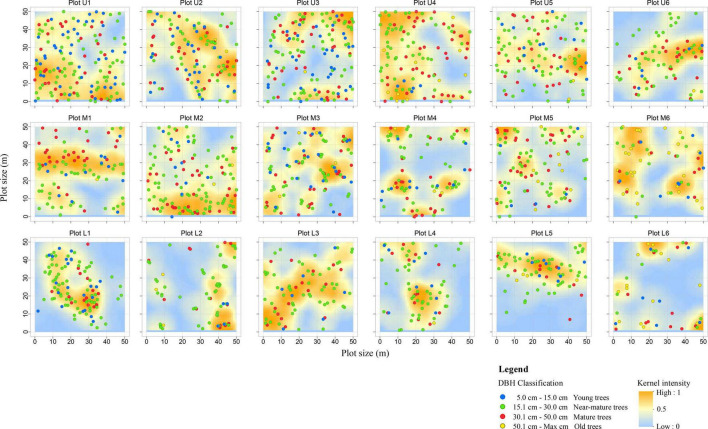
Spatial distribution of *P. euphratica* trees at each (50 m × 50 m) plot within four DBH classes in the 18 plots. The first column (Plot U1–U6) represented the upper reaches, the second column (Plot M1–M6) represented the middle reaches, and the third column (Plot L1–L6) represented lower reaches plots. Every point of a different color indicates the position of individual trees at a different DBH class. The change of background color indicates kernel intensity map. The deep yellow indicates a highly concentrated area; the pale yellow indicates a scattered area; and the blue indicates a low concentrated area.

Individual *P. euphratica* tree locations were recorded and acquired kernel intensity map for each plot based on the TLS data (Figure 5). Trees are more evenly distributed at plots U1–U5 (upper reaches) and M2–M3 (middle reaches), while the trees of the remaining plots in the middle and lower reaches are unevenly distributed. Young and near mature trees are concentrated in plots were close to the river channel (Figure 5); mature trees are evenly distributed in each plot; and old trees are generally located at distances far from the river channel (e.g., plots M5–M6 and L5–L6).

The Kernel density of the spatial distribution patterns reveals a random distribution for the trees in the upper reaches; in the middle reaches, the trees are typically distributed in aggregated patterns inside the plot areas, with random patterns outside the aggregated area; and the majority of trees in the lower reaches exhibit an aggregated distribution, with young and near mature trees distributed around the mature trees (Figure 5).

**FIGURE 6:**
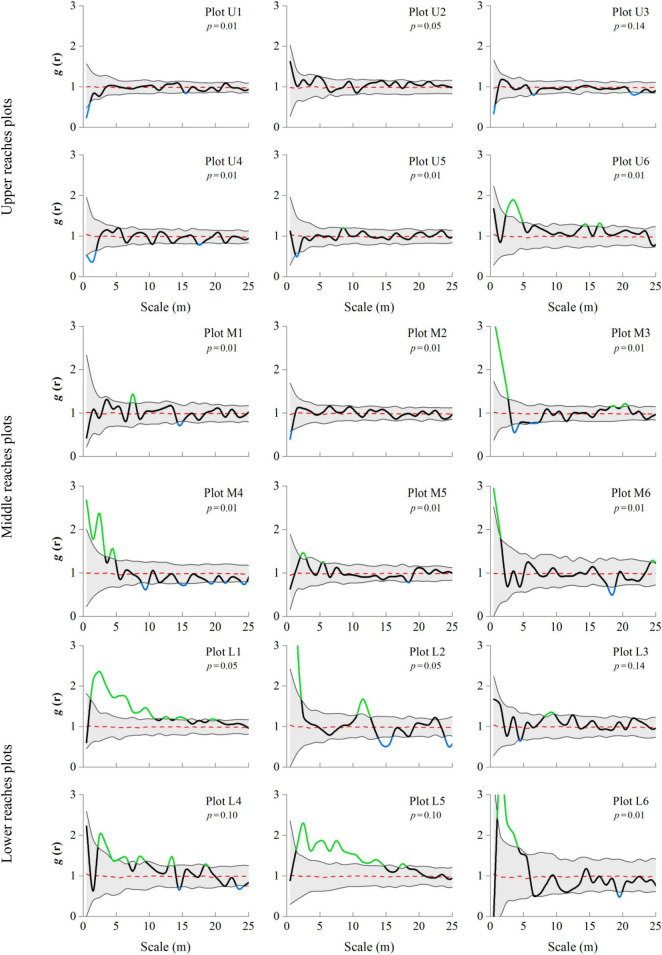
Spatial distribution patterns of *P. euphratica* trees across different river transects. Representation of pair correlation g(*r*) analysis using complete spatial randomness (CSR) null models assessed all forest plots. Bold lines indicate the g(*r*) function value, green indicates values above CSR model confidence envelopes (aggregated), blue indicates values below envelopes (regular), and black indicates values within the envelopes (random), and shaded areas indicate the Monte Carlo envelopes 99% confidence interval.

Variations are observed in the distribution patterns (Figure 6). The point pattern analysis based on the g(*r*) function under the CSR null model reveals upper reaches plots U1–U5 [g(*r*) ≈ 1] to exhibit a random pattern, while plot U6 (located 1000 m away from the river channel) is associated with an aggregated pattern. In the middle reaches, the distribution presents a random pattern [g(*r*) ≈ 1] for plots M1 and M2, which are located close to the river channel, and an aggregated pattern [g(*r*) > 1] for plots M3–M6. The distribution of the lower reaches generally exhibits an aggregated pattern [g(*r*) > 1], except for plot L3. The highest degree of aggregation is observed for plots L2 and L6 [g(*r*) = 9.7, 3.7] in the lower reaches, followed by plots M3 and M6 [g(*r*) = 3.1, 2.2] in the middle reaches, and plot U6 [g(*r*) = 1.8] in the upper reaches (Figure 6). The g(*r*) function average value exhibited the following order: lower reaches [g(*r*) = 1.3] > middle reaches [g(*r*) = 1.1] > upper reaches [g(*r*) = 1.0], indicating that the main distribution pattern of trees from the upper to lower reaches has changed from random to cluster distribution pattern.

Table 3 showed that the ANN index values are high at the upper reaches ranged from 0.95 to 1.18, except the plot U4 and U6, other plots indicating a generally random distribution; values of the middle reaches range from 0.72 to 1.06, indicating a distribution that changes from random to aggregated pattern; and the lower reaches values range from 0.54 to 0.94, revealing an aggregated pattern, except plot L3 which showed random pattern. The DMI, HIS and *I*_δ_ index results also same as the ANN index, at the 0.05 significant levels. These observations agree with g(*r*) function result.

**TABLE 3:** Spatial distribution pattern index of ANN, DMI, HIS, and I_δ_ analysis results of *P. euphratica* in the three river transects.

Plots	ANN	*z*-score	Distribution pattern	DMI	*t*-value	Distribution patterns	HSI	*x*-value	Distribution patterns	I_δ_	*t*-value	Distribution patterns
U1	1.08	4.22**	R	0.86	–0.34	U	0.52	0.30	U	1.01	–0.25	R
U2	0.97	−0.56**	R	1.24	0.54*	R	1.16	0.51	A	1.12	2.72**	R
U3	1.06	1.33*	R	1.17	0.40*	R	0.78	0.44	U	0.99	–0.17	R
U4	1.23	4.71**	U	0.95	0.40*	U	0.48	0.32	U	1.02	0.38	R
U5	1.03	2.83**	R	0.76	–0.55	R	0.55	0.35	U	0.98	–0.34	R
U6	0.95	–0.80	A	2.20	2.49**	A	1.09	0.52*	R	1.68	4.56**	A
M1	1.06	1.03	R	1.27	0.58*	R	0.97	0.49*	R	0.99	0.89	R
M2	1.06	1.35	R	1.13	0.31*	R	0.67	0.40	U	1.06	1.13	R
M3	0.72	−5.76**	A	1.42	0.96**	A	1.62	0.61**	A	1.72	1.11	A
M4	0.74	−4.65**	A	2.33	2.85**	A	1.67	0.62**	A	1.99	4.43**	A
M5	0.97	–0.64	R	1.93	0.58*	A	1.31	0.57*	A	1.38	0.97	A
M6	0.81	−2.82**	A	1.29	2.08**	A	3.68	0.79**	A	1.17	2.32**	A
L1	0.86	−2.78**	A	3.33	5.23**	A	1.47	0.60**	A	1.89	12.33**	A
L2	0.54	−7.50**	A	2.03	2.11**	A	1.23	0.55**	A	1.46	4.69**	A
L3	0.94	–0.97	R	1.25	0.52*	R	1.12	0.53	A	1.08	0.88	A
L4	0.87	−1.99*	A	1.88	1.76**	A	1.22	0.55**	A	1.44	3.99**	A
L5	0.87	−2.20*	A	3.72	5.61**	A	0.65	0.39	U	1.72	6.06**	A
L6	0.80	−2.33*	A	2.01	1.83**	A	0.72	0.42	U	1.99	5.29**	A

R represented random pattern; U represented uniform/regular pattern; A represented aggregated pattern.** was significantly correlated at 0.01 level; * significantly correlated at 0.05 level.

### Effects of Abiotic Factors on the Spatial Distribution Patterns

The correlation between the different abiotic factors of the plots, including the groundwater depth (GD), soil moisture (SM), soil conductance (SC), soil pH value and distance from the river channel (DR), and ANN index was assessed (Table 4). The GD, SM, and SC correlate with the ANN index.

**TABLE 4:** Correlation values between ANN and abiotic factors.

	DR	GD	SM	SC	pH
ANN	−0.096	0.671**	0.569*	−0.538*	−0.291

** was significantly correlated at 0.01 level; * significantly correlated at 0.05 level.

**FIGURE 7:**
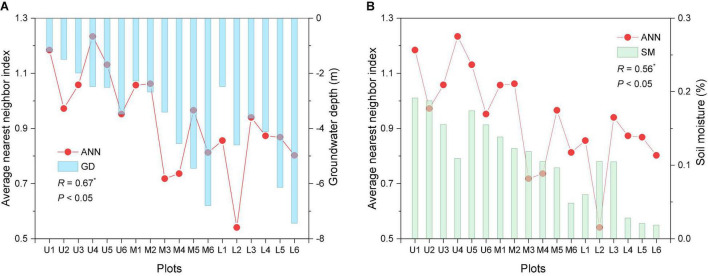
Spatial pattern index, GD, and SM at all transects. ANN is the nearest neighbor index; GD is the groundwater depth; SM is the soil moisture; *R* is the correlation coefficient; * represents significant correlation (*P* < 0.05). On X-axis the U1–U6, M1–M6, and L1–L6 represented the upper, middle, and lower reaches plots.

The average GD and SM values exhibit a decreasing trend from the upper to lower reaches (Figure 7). Correlation analysis reveals the ANN index to be significantly positively correlated with GD and SM (*R* = 0.67 and 0.56, respectively, *P* < 0.05).

## Discussion

### Terrestrial Laser Scanning Determined Spatial Distribution of Riparian Forest

Various traditional field measurement methods have been used for analyzing the spatial distribution pattern of riparian forests (Han et al., 2014; Xu et al., 2016; Miao et al., 2020). However, traditional methods are hard to obtain detailed structural parameters, and spatial distribution patterns of the forest stand in the same period, which hindering our deeper understanding of forest structure. As an active remote sensing method, LiDAR has obvious advantages for determining forest structural parameters (Guo et al., 2018; Calders et al., 2020). In this study, we used TLS data for the first time on riparian forests and demonstrated that the LiDAR techniques have high advantages for measuring the desert sparse forest type (Figure 3). TLS can quickly and accurately determine forest structure attributes in different stand density plots. We comparatively analyzed the stand structure parameters, population dynamics, and spatial distribution pattern of *P. euphratica* forest in different transects of the Tarim River.

### Structural Characteristics Variations of *Populus euphratica*

The number of trees, SDI, CPA, and TH decreased significantly in our study sites from the upper to lower reaches of the Tarim River (Table 2). This indicated that the structural characteristics of *P. euphratica* forest have changed in the different sections of the Tarim River. For example, the upper transect exhibited the highest average TH and lowest average DBH, while the CPA values exceeded 80%, demonstrating the high vitality of the forests (Han et al., 2017). The TH in the middle reaches less than the upper reaches, while the average DBH was the largest, with CAP values higher than 60%, indicating a relatively good forest growth trend. In the lower reaches, the average TH and DBH were lower than the middle reaches, and the CPA was smaller than 40%, revealing that the forests were degraded. Previous studies have reported the individual growth of *P. euphratica* to adapt to deteriorated environments by adjusting the size of the CD and reducing the TH (Yang et al., 2014; Aishan et al., 2015). The growth of *P. euphratica* highly depends on the groundwater and soil moisture (Halik et al., 2009; Keyimu et al., 2017a). Under extreme drought stress, morphological characteristics of trees shift the form to extend their survival period to wait for a favorable change (Miao et al., 2020). Monda et al. (2008) demonstrated near mature *P. euphratica* trees at the Heihe River to exhibit damage at the top part of the crown, while the dieback phenomenon was observed, suppressing their height. Trees can survive increasingly dry environments by abandoning assimilation organs, thereby suppressing height growth as a price for survival (Xu et al., 2016). The higher canopies above thick trunks and wider leaves of *P. euphratica* become disadvantageous under severe drought stress (Li et al., 2017). In addition, the increased intensity of inter-species competition reduces the density and survival rate of the *P. euphratica* population, significantly changing structural indicators such as TH, DBH, and CD (Shi et al., 2021). Therefore, the change of stand structural attributes and growth status of the *P. euphratica* indicated the adaptation to different environmental conditions.

### Population Structure of *Populus euphratica* in Different Transects

In harsh dry conditions, the growth potential of the *P. euphratica* decreases while the mortality rate increases (Chen et al., 2014; Keram et al., 2021). Thus, the population structure evolves from a growing to a declining population, and the forest stand gradually changes from a juvenile to an over mature forest (Han et al., 2017). Our result indicated distinct population structure characteristics across the upper, middle, and lower reaches of the Tarim River (Figure 4). The plots of the upper reaches exhibited a DBH class structure that followed a logistic fit, demonstrating a growing population trend, while the middle and lower reaches plots followed a Gaussian fit, with a stable population trend. However, the scarcity of young trees leads to a weak anti-disturbance ability of the forest population in the lower reaches, and thus the renewal and rejuvenation of forests are difficult under natural conditions. Han et al. (2014) found the young tree ratio in the upper reaches of the Tarim River to be high, corresponding to the Deevey I type survival curve (Deevey, 1948). This indicated an expanding population trend. Under suitable environmental conditions, shallow groundwater depth during the flood season, which coincides with the dispersal period of *P. euphratica* seeds, enables the production of more seedlings in the forest. The DBH class exhibited a normal distribution in the middle reaches, with relatively few young trees and more mature trees, demonstrating a stable population trend (Xu et al., 2016; Han et al., 2017). In the lower reaches, Keyimu et al. (2017b) and Zhou et al. (2018) found the mature trees to occupy a large population ratio. Therefore, the living conditions must be improved to enhance the survival rate and the number of young *P. euphratica* trees in the middle and lower reaches of the Tarim River. The water resources in the three reaches of the Tarim River are unevenly distributed across time and space, and the gradually declining groundwater level from the upper to lower reaches consequently resulted in different population structures in the respective transects.

### Changes in the *Populus euphratica* Spatial Distribution Patterns

Our results reveal that the *P. euphratica* forests exhibit varying spatial patterns in the three transects of the Tarim River (Figure 5). In the upper reaches, the spatial pattern index g(*r*) function value was close to 1 and fluctuated within the CSR null model confidence interval, representing a random distribution pattern (Figure 6). This was also true for the ANN, DMI, and *I*_δ_ indexes (Table 3). These observations agree with previous work findings reported by Zhang et al. (2019) and Miao et al. (2020), which showed that in the upper reaches of the Tarim River, the *P. euphratica* live trees were typically randomly distributed. The trees in the middle reaches were unevenly distributed, except for two plots (M1 and M2) closest to the river (Figure 5), with the g(*r*) function producing high values above the CSR null model confidence interval. Moreover, the ANN index demonstrated a decreasing trend, implying that the spatial distribution changed from random to aggregated patterns with increasing distance to the river channel. Wang et al. (2020) found that the old tree ratio increased with the distance from the river channel, and the distribution pattern changed. Zeng et al. (2019) and Zhang et al. (2019) reported the aggregation intensity of the Morisita index to increase from the upper to the middle reaches of the Tarim River. Yu et al. (2021) revealed that the *P. euphratica* trees in the lower reaches of the Heihe River to be randomly distributed within a 100 m distance from the river channel and aggregation intensity was increase as increasing the distances from the river. These results are consistent with the current study (Table 3). The plots (L1∼L6) in lower reaches exhibited g(*r*) function values above the CSR confidence interval, and ANN index values less than 1 indicated the aggregated pattern. Moreover, the Kernel intensity showed strong cluster intensity in a partial area within the plots (Figure 5). These results are consistent with previous work by Zhou et al. (2018) and Zhang et al. (2019). *P. euphratica* forest aggregation intensity generally increased from the upper to lower reaches, and the spatial distribution changed from random to aggregated pattern.

### Response of Spatial Distribution Patterns to Heterogeneous Environmental Conditions

Spatial pattern differences result from the interaction between plants and abiotic factors, particularly the biological plant characteristics, the competition between species, and the habitat environment (Li and Zhang, 2015; Podlaski, 2019). In arid regions, water from rivers infiltrates into the riparian groundwater (Westermann et al., 2008). River diversion and flooding are the main factors influencing the dominance of the Tarim River basin in global *P. euphratica* forest locations (Zhang et al., 2019). *P. euphratica* trees are phreatophytes and thus rely on groundwater to survive (Thomas and Lang, 2020). The spatial heterogeneity of the water environment induces changes in the morphological and physiological characteristics of *P. euphratica* (Shi et al., 2021). Ling et al. (2015) determined the minimum groundwater depths for young, near-mature, mature, and old trees as 4.0, 5.0–5.4, 6.9, and 7.8 m, respectively. Moreover, the groundwater level exhibits a descending gradient from the upper to lower reaches (Chen et al., 2006; Zeng et al., 2019). The soil moisture has also been observed to decrease gradually from the upper to lower reaches of the Tarim River (Han et al., 2016), thus resulting in differences in the growth and distribution of *P. euphratica* (Gries et al., 2003; Xu et al., 2016; Zhang et al., 2019). The survival and growth rate of *P. euphratica* decreased with the groundwater level (Aishan et al., 2015, 2018; Ling et al., 2015, 2017; Yu et al., 2021). Our results revealed a significant positive correlation between the spatial pattern index ANN and values of GD and SM (Figure 7). Furthermore, reductions in the groundwater level and soil moisture content were associated with an increase in the aggregation intensity of spatial distribution patterns. In the upper reaches plots, the shallow groundwater level and relatively abundant soil moisture conditions contributed to maintaining a high forest stand density (Table 3) and growing population structure (Figure 4), and thus the spatial distribution was generally formed random pattern. Miao et al. (2020) found the riparian forest growing in an area of the Daliyaboyi Oasis with a groundwater depth of 3–5 m to present a random distribution. In the middle and lower reaches of the Tarim River, the GD and SM decreased with increasing distance from the river (Figure 7), and consequently, the ANN index values also exhibited a decreasing trend.

## Conclusion

The TLS method can quickly and accurately obtain 3D spatial structure with high resolution under different stand densities and provides repeatable application and processing, which improves the shortcomings of traditional forest inventory. The TLS-derived results revealed that the stand structure attributes, population age structure, and spatial distribution pattern of *P. euphratica* forest in the three transects of the river have obvious differences. The forest density, coverage, and growth performance were reduced from the upper to the lower reaches, and population age structures revealed a growing trend in the upper reaches, a stable trend in the middle reaches, and a temporarily stable trend in the lower reaches of the Tarim River. The spatial distribution pattern of forests changed from random pattern in the upper reaches to random and aggregated pattern in the middle reaches and aggregated pattern in the lower reaches.

Groundwater, soil moisture, and soil salinity conditions are the key abiotic factors affecting the germination of seedlings, and the development and spatial distribution of riparian forests. The *P. euphratica* trees typically exhibit a random distribution pattern in regions where groundwater fluctuations are not significant. Groundwater lower than suitable level is located in the areas far away from the rivers, thus reducing tree density, tree growth, and inducing an unstable population structure, and aggregated spatial distribution pattern.

Based on our findings, we put forward following suggestions to promote the population structure and spatial distribution pattern of *P. euphratica* in the middle and lower reaches of the Tarim River. First, the reasonable allocation of water resources along the Tarim River must be achieved to balance the water requirements by agriculture and ecology among the upstream, midstream, and downstream regions. Second, floods are important in the formation of *P. euphratica* riparian forests as they transport the seeds to germination sites and replenish the groundwater and soil moisture allowing the seeds to germinate. Therefore, artificial hydrological alterations on water resources must be implemented by digging ecological irrigation channels and expanding the overflow area *via* manual regulation of water resources during the germination period of *P. euphratica* seeds in the middle of July to the middle of August. Our study limitations were that only one scale plot size measured by TLS and did not consider the stand distribution patterns at a large scale, which may impact the research results and further research is needed in the future.

## Data Availability Statement

The original contributions presented in the study are included in the article/Supplementary Material, further inquiries can be directed to the corresponding author.

## Author Contributions

AY and ÜH conceived, designed, and performed the experiments, collected and analyzed the data, prepared figures and tables, and wrote the manuscript draft. AA, TA, and MK participated in field work and contributed to data collection, analysis, and the manuscript writing. JW read the manuscript with critical comments and reviewed the manuscript draft. All authors checked and approved the final content of the manuscript.

## Conflict of Interest

The authors declare that the research was conducted in the absence of any commercial or financial relationships that could be construed as a potential conflict of interest.

## Publisher’s Note

All claims expressed in this article are solely those of the authors and do not necessarily represent those of their affiliated organizations, or those of the publisher, the editors and the reviewers. Any product that may be evaluated in this article, or claim that may be made by its manufacturer, is not guaranteed or endorsed by the publisher.
