# Supplementary material for: Population Structure and Spatial Distribution Pattern of Populus euphratica Riparian Forest Under Environmental Heterogeneity Along the Tarim River, Northwest China
Source: Front Plant Sci. 2022 Jun 16;13:844819. doi: 10.3389/fpls.2022.844819 (PMC9244701; doi:10.3389/fpls.2022.844819)
Supplement: Supplementary file 1 [file Data_Sheet_1.pdf]

Highlights:

1. This is the first successful application of Terrestrial Laser Scanning (TLS) for detecting the tree morphological structure of *Populus euphratica* riparian forests along the Tarim River.
2. We confirmed that the population dynamics of riparian forests was growing in the upper reaches, stable in the middle and lower reaches.
3. *P. euphratica* trees were distributed in random, random to aggregated, and aggregated patterns in the upper, middle, and the lower reaches, respectively.
4. Recommendations for local stakeholders to optimize their forest management strategy were put forward.
