# Supplementary material for: Population Structure and Spatial Distribution Pattern of Populus euphratica Riparian Forest Under Environmental Heterogeneity Along the Tarim River, Northwest China
Source: Front Plant Sci. 2022 Jun 16;13:844819. doi: 10.3389/fpls.2022.844819 (PMC9244701; doi:10.3389/fpls.2022.844819)

Detection of population structure and spatial distribution patterns of floodplain forests using TLS

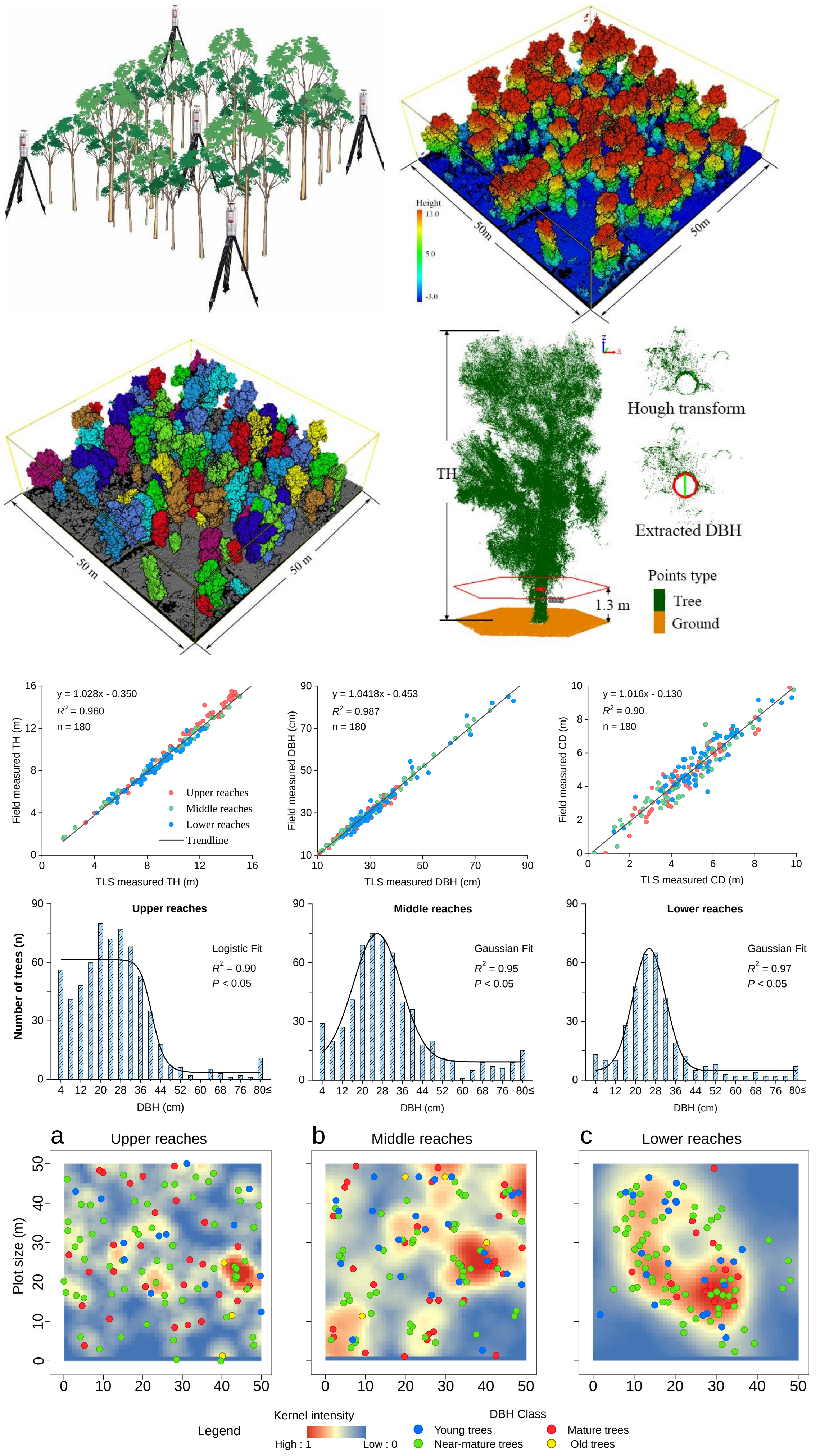

Supplement: Supplementary file 2 [file Data_Sheet_2.pdf]
